# An examination of domestic partner violence and its justification in the Republic of Georgia

**DOI:** 10.1186/1472-6874-13-44

**Published:** 2013-11-01

**Authors:** Eve Waltermaurer, Maia Butsashvili, Nata Avaliani, Steve Samuels, Louise-Anne McNutt

**Affiliations:** 1 Department of Sociology, State University of New York, New Paltz, NY 12561, USA; 2 National Center for Disease Control and Public Health, Tbilisi 0177, Georgia; 3 School of Public Health, Department of Epidemiology and Biostatistics, One University Place, GEC Building, Rensselaer, NY 12144, USA

**Keywords:** Partner violence, Social norms, Women, Gender, Republic of Georgia

## Abstract

**Background:**

Little research on Intimate Partner Violence (IPV) and social perceptions toward this behavior has been disseminated from Eastern Europe. This study explores the prevalence and risk factors of IPV and the justification of this behavior among women in the Republic of Georgia. It seeks to better understand how IPV and IPV justification relate and how social justification of IPV differs across socio-economic measures among this population of women.

**Methods:**

This study utilizes a national sample of ever-married women from the Republic of Georgia (*N* = 4,302). We describe the factors that predict IPV justification among these women and the relationship between of the acceptability of IPV and victimization overall and across socio-demographic factors.

**Results:**

While the overall lifetime prevalence of IPV in this sample was relatively low (4%), these women were two to four times more likely to justify IPV, Just under one-quarter of the sample agreed that IPV was justified in at least one scenario, namely when the wife was unfaithful, compared with women who had no experience being abused by a partner. Georgian women who were poor, from a rural community, had lower education, were not working and who experienced child abuse or IPV among their parents were more likely to justify this behavior.

**Conclusions:**

These findings begin to fill a gap in our understanding of IPV experienced by women in Eastern Europe. In addition, these findings emphasize the need for researchers, practitioners and policy makers to contextualize IPV in terms of the justification of this behavior among the population being considered as this can play an important role in perpetration, victimization and response.

## Background

With little to no published research on intimate partner violence disseminated from Eastern Europe, this study pays particular focus on IPV and the social justification of this behavior among women from Georgia. Georgia has experienced a tremendous amount of social and economic turmoil in the past two decades following its independence from the Soviet Union in 1991. Upon gaining sovereignty, Georgia has undergone several armed conflicts resulting in splitting the country and establishing de-facto separated regions of South Ossetia and Abkhazia. Disagreement over reforms resulted in violent conflicts and ethnic cleansing. Furthermore, socioeconomic changes during the intervening decades have created an environment conducive to increasing IPV. Prior to the upheaval Georgia experienced relative income equality, but after 1990 increased inflation drove the poverty rate up. Although the United Nations Commission on Humans Rights adopted their Declaration on the Elimination of Violence against Women in 1993 [1], it was not until 2006 that Georgia instituted its first law on domestic violence and defined it to include acts of physical, psychological, economic and sexual violence between family members. Furthermore, domestic violence laws in Georgia are argued to be vague as they are enveloped into general criminal codes against violence, tending to ignore domestic violence as a unique condition and paying no regard to psychological violence [2]. Interestingly, the IPV data that have been reported show the prevalence in Georgia as being notably low -- fewer than 8% of women report ever experiencing any victimization [3,4]. For context, a multi-county study on women’s health and domestic violence in ten countries between the years of 2000 and 2003 conducted by the World Health Organization (WHO) obtained IPV prevalence rates ranging from 15%-71% with only two countries with a prevalence of under 25% [5]. This lower IPV prevalence in Georgia seems to contradict research identifying a typically higher rate of IPV in countries highly impacted by conflict [6,7].

While there have been high levels of conflict experienced by the populace, Georgians are more often the victims than the oppressors. Yet on an individual level, gender power differentials have been featured in Georgia’s past including bridal kidnappings where men would kidnap virgin women to rape them in order to keep them from marrying any man except for the offender [8]. Given these experiences, it is interesting to contextualize IPV in Georgia within a larger framework of social justification of this behavior. The examination of partner violence justification and its impact on IPV victimization has seen some growth internationally [5,9-13]. These studies have found that the rate of justification in many countries are quite high and can vary by the reason for abuse (e.g. neglect of child, infidelity). Furthermore, these studies found that women tended to approve of IPV at a greater rate than males and factors reflecting lower socio-economic status resulted in typically higher acceptance of IPV.

Drawing from a small but growing body of international research, conducted primarily over the last decade, a clearer understanding of the interaction between social justification of partner violence and its incidence has developed. “Injunctive” social norms, or consensus about IPV being acceptable or not within a community [12,14] recognizes that while partner violence often occurs in the privacy of one’s home, it is informed by the attitudes of the larger society. Furthermore, cultural differences in the incidence of partner violence has been argued as a reflection the attitudes shared by the group that governs interactions within each culture [15]. The conceptualization of the intersection between social norming and IPV is integrated within the earliest conceptualizations of this problem. Two of the most often cited theoretical frameworks applied to understand or distinguish types of partner violence are common couple violence theory and the theory of patriarchal terrorism; each conceptualization centers strongly on the role of social perceptions of violence and/or women [16,17].

The common couple violence theory posits that general social attitudes toward violence are central to producing a more violent society where IPV can exist [18]. While the core of this framework is on partner violence being gender symmetric, an argument that stems from the idea that behaviors of both males and females reflect that of their larger culture’s endorsement of violence, these general attitudes about the acceptability or non-acceptability of violent behaviors can influence the justification of partner violence in subtle ways. A key element of the common couple violence framework includes the nature of what behaviors we define as violent, for example lower threshold violence may encompasses greater female perpetrators while acts of domestic homicide primarily are perpetrated by males. In one British study it was found that there was not full agreement as to what behaviors constituted partner violence finding that 16% of urban participants did not feel slapping denoted partner violence and 5% did not feel getting punched was an act of partner violence [19]. Clearly if a behavior is not seen as violent, it will be deemed more acceptable by those who experience, perpetrate and respond to it.

The patriarchal terrorism theory points to social attitudes around the role of women in relation to men as the source of partner violence [20]. The role of patriarchy within a culture plays an intricate role in social perceptions of partner violence toward women as it can support attitudes that men are not responsible for their behaviors whereas women are to blame [21], women’s behaviors are the triggers of violence by partners [22], and that women secretly desire this exertion of power [9]. These attitudes interchange with the level of tolerance an individual will feel toward IPV which in turn can play an important role in influencing whether these violent acts are reported to a third party such as the police [23,24].

Examples of patriarchal hegemony in Georgia do exist. While the actual prevalence is not known, there was a time when bridal kidnapping, where single men would kidnap virgin women to rape them in order to keep them from marrying any man except for the offender were not uncommon [8]. Yet, for over a decade, the rate of reported partner violence is Georgia is consistently low [3,4]. Georgia provides an interesting case study as it conceptually represents a society like the U.S and Europe of the past in which women may be viewed as inferior, but families are often intact and multigenerational, and the influences of the western world are only recently emerging. Within this context it is further relevant to understand the role of socio-demographics such as age, geography, marital status, education, work status and wealth play in this dynamic as we recognize these factors influence IPV consistently across the globe [25].

To further explore the relationship between IPV victimization and the acceptance of this behavior among Georgian women, our study takes advantage of the justification scenarios built into the most recent Women’s Reproductive Health Survey in Georgia (2010). To better understand the factors associated with the justification of partner violence in Georgia, this study seeks to answer the following questions: 1) What factors are associated with Georgian women justifying partner violence against women? and 2) To what extent does experiences with domestic violence predict IPV justification?

## Methods

### Dataset

This study is a secondary data analysis of the 2010 Women’s Reproductive Health Survey (RHS) in Georgia (GERHS10). Permission to use this data set came from the Georgian National Center for Disease Control (NCDC). The principle purpose of the original RHS is to examine factors related to pregnancy and fertility among Georgian women. Previous RHSs in Georgia took place in 1999 and 2005. Surveys are conducted by the Georgian Ministry of Labor, Health and Social Affairs (MoLHSA) in collaboration with the Georgian National Center for Disease Control (NCDC) Division of Reproductive Health, Centers for Disease Control and Prevention (DRH/CDC), the United Nations Population Fund (UNFPA), and the United States Agency for International Development (USAID). These surveys utilize large nationally representative probability samples of women aged 15–44 years. Women are interviewed in their homes. A total of 6,292 women were interviewed in 2010. For further details, refer to the RHS summary report [4]. Only those women who were ever married were asked about justification for IPV (i.e., is a man ever justified for beating his wife). Thus, the sample studied here was restricted to the ever-married women. In addition, with the focus on IPV justification, women were excluded if they answered “don’t know” or had a missing response for any of the justification scenarios as these measures were dichotomized and aggregated (see below). We also excluded women who had a missing response to any of the past or current domestic violence experience questions to allow our raw totals for the prevalence estimates to be the same. These exclusions resulted in the loss of only 185 women (4% of the data) and resulted in a final sample of 4,301. This sample consisted of mostly currently married women between the ages of 24 and 44. This human subject research study was in compliance with the Helsinki Declaration approved by the Institutional Review Boards (IRBs) of both the Georgian National Center for Disease Control and Public Health and the United States Center for Disease Control (CDC).

### Measurements

Included demographic measures are those factors identified as typically associated with intimate partner violence risk through a World Health Organization (WHO) multi-country study [25]. These include: age (15–24, 25–34, 35–44), residence (rural, urban); marital status (not currently married, currently married); education (did not complete secondary, completed secondary only, completed university or technical college); and working status (yes, no). Wealth (low, middle, high) was measured as composite index based on ownership of a television, automobile, refrigerator, videocassette recorder, cell phone, land-line phone, flush toilet, heating system, vacation home and having more than one room per household member.

The experience of IPV was measured using the modified eight-item Conflict Tactic Scale [26], which includes acts of physical, psychological (e.g., insults and threats of harm) and sexual abuse of ever-married women during her lifetime and within the past year. This study focused on “prior to past year” and “past year” reports of physical violence only which included being pushed, shoved, slapped, kicked, hit with the fist or an object, beaten up, and threatened with a knife or other weapon. While lifetime and past year partner violence is commonly explored as distinct outcomes, since these were both included in the same model as covariates, this produces issues with collinearity. As a result, those who experienced violence in the past year, with or without exposure before were classified within “past year” while those who reported lifetime abuse, but none in the past year were classified within “prior to past year.” In addition, experience with domestic violence as a child (yes/no) was measured using two questions. The first asked the respondent if, as a child or adolescent, she had witnessed physical abuse between her parents; this first question was only asked to women who reported being raised in a household with both parents. The second asked the respondent if she experienced abuse from her parents as a child or adolescent.

Partner violence justification was measured in the GERHS10 by assessing agreement (yes/no) to seven scenarios. Specifically, women were asked if they felt a husband would be justified in beating his wife if: 1) she goes out without telling him; 2) she neglects the children; 3) she argues with him; 4) she refuses to have sex with him; 5) he is not happy with her household work or food provisions; 6) she asks him whether he has other girlfriends; 7) he finds out that she has been unfaithful. For this study, each scenario is examined separately. In addition, responses are dichotomized to those who responded affirmatively to any one scenario compared with those who responded negatively to all. This “any justification” variable was created to allow for comparisons across other similar studies which utilize this grouped measure.

### Statistical analysis

Prevalences were weighted, adjusting for sample design. Weighted prevalence’s were calculated by Stata's “SVY: TABULATE” command, which employs the standard formula for weighted means [27]. Descriptive statistics, including frequencies and percentages, were computed and tested using categorical chi-square tests for statistical significance between measured factors and justification of IPV in any scenario as well as between IPV victimization status and each specific IPV justification scenario. Prediction models were created for each scenario and the combination of any scenario of IPV justification using a survey log binomial regression to estimate crude and adjusted prevalence ratios. For each adjusted model all measured covariates were included except where noted in the table. Exclusions of covariates were only made when the models that included those variables failed to converge. Log-binomial was utilized as the odds ratio generated through logistic regression tends to overestimate prevalence among common outcomes [28].

## Results

### Demographics

The total sample of 4,302 ever married women aged 15 to 44 years included primarily women over 25 (Table 1). There was a relatively equal distribution across wealth tercile, and just over half of the women reside in a rural area. While this sample includes only women who were ever married, a few (9%) were not currently married. Over half of the women had completed university or technical college; however, over three quarters were not working. Fewer than 9% reported ever witnessing IPV among their parents or experiencing abuse as a child. Just under 4% reported experiencing IPV victimization prior to the past year and close to 2% reported past year IPV victimization. Significant differences in the justification of IPV overall were apparent across each socio-demographic measure except for age and partner violence history.

**Table 1:** Sample descriptives ever married women of reproductive age in country of Georgia

		**n**	**% of total**	**% agree with at least one scenario**	**p**
Total		4,302		19.1	
Age	15-24	728	16.9	20.8	0.06
	25-34	1,864	43.3	17.1	
	35-44	1,710	39.8	20.3	
Wealth	Low	1,389	32.3	27.0	<0.01
	Middle	1,566	36.4	18.8	
	High	1,347	31.3	13.3	
Residence	Rural	2319	53.9	24.5	<0.01
	Urban	1983	46.1	14.0	
Marital status	Not currently married or in union	374	8.7	10.1	<0.01
	Currently married or in union	3,928	91.3	20.1	
Education	Did not complete secondary	743	17.3	25.5	<0.01
	Completed secondary only	1,152	26.8	23.5	
	University or Technical College	2,407	55.9	15.0	
Work	Not working	3,278	76.2	20.8	<0.01
	Working	1,024	23.8	13.5	
Parental IPV	Yes	366	8.5	31.1	<0.01
	No	3,936	91.5	17.9	
Abused as a child	Yes	366	8.5	35.0	<0.01
	No	3,936	91.5	17.7	
Partner violence history	None	4,090	95.1	19.0	0.35
	Prior to past year only	145	3.4	17.8	
	Past year	67	1.6	27.1	

Note: Limited to women who were ever married as these were the only ones asked about IPV justification. The general sample lifetime prevalence is 7%.

Overall just over 19% of the Georgian women in this sample agreed that a husband is justified in beating his wife in at least one scenario. This was primarily driven by the scenario: “when a wife is unfaithful” which justified IPV according to 18.6% of the sample. The next remaining scenarios were viewed as cause for IPV by five percent or less of the women, specifically and in order of increased prevalence: when a wife neglects her children (5.0%); when a wife argues back (3.3%); when a wife asks about other girlfriends (2.4%); when a wife goes out without her husband’s permission (1.8%); when a wife refuses sex (1.6%) and when a wife neglects housework (1.3%).

### Predicting justification of intimate partner violence

Predictive models were examined to better understand the factors most associated with justification of IPV under at least one circumstance as well as across specific circumstances for Georgian women. Tables 2, 3 and 4 presents both crude and adjusted results as in many cases, the adjusted models resulted in a factor showing no significance due to sparse cells. Table 2 focuses on the associations between socio-economic factors on the justification of IPV. It was found that, women in the lowest household income tercile were two to twenty-four times more likely to justify IPV when compared with women of the highest income depending on the scenario. In terms of education, the lower a woman’s education, the higher the likelihood that she would justify partner violence across each scenario. Lastly, women who were not currently working consistently showed a higher likelihood to justify IPV across each scenario when compared with women who were currently working.

**Table 2:** Socio-economic predictors of IPV justification

	**Wealth (High referent)**				**Education (University/Tech College referent)**				**Employment (Currently working referent)**	
	**Low**		**Medium**		**Did not complete secondary**		**Completed secondary**		**Not working**	
	**Crude**	***Adjusted***	**Crude**	***Adjusted***	**Crude**	***Adjusted***	**Crude**	***Adjusted***	**Crude**	***Adjusted***
Any	2.04 (1.59-2.62)	*ns*	1.42 (1.09-1.84)	*ns*	1.70 (1.31-2.21)	*1.31 (1.02-1.69)*	1.57 (1.32-1.87)	*1.33 (1.09-1.62)*	1.54 (1.23-1.92)	*ns*
Out w/out permission	5.88 (2.00-17.23)	*ns*	ns	*ns*	8.58 (3.81-19.36)	*4.41 (1.95-9.96*)	2.80 (1.22-6.44)	*ns*	8.90 (2.50-31.74)	*3.72 (1.03-13.47*)
Neglects kids	3.72 (2.20-6.30)	*1.99* (*1.06-3.75*)	1.81 (1.04-3.16)	*ns*	4.05 (2.59-6.32)	*2.68 (1.66-4.33*)	2.03 (1.37-3.01)	*1.63 (1.03-2.59*)	2.34 (1.41-3.87)	*ns*
Argues back	4.61 (2.23-9.52)	*ns*	ns	*ns*	7.29 (3.87-13.75)	*4.60 (2.48-8.51*)	2.84 (1.60-5.05)	*2.03 (1.07-3.85*)	5.00 (2.39-10.48)	*2.49 (1.11-5.60*)
Refuses sex	9.12 (2.43-34.22)	*ns*	ns	*ns*	15.46 (4.75-50.29)	*6.75 (1.86-24.46*)	5.24 (1.68-16.33)	*ns*	39.53 (5.09-306.78)	*13.46 (1.67-111.28*)
Housework/Food prep	23.92 (5.17-110.70)	*8.58 (1.80-40.94*)	7.86 (1.52-40.72)	*ns*	16.80 (5.71-49.43)	*7.65 (2.61-22.46*)	4.08 (1.46-11.41)	*ns*	*	*
Ask about girlfriends	ns	*ns*	ns	*ns*	4.52 (1.77-11.56)	*3.31 (1.49-7.32*)	3.49 (1.74-6.98)	*2.86 (1.36-6.01*)	6.56 (1.96-21.94)	*4.53 (1.26-16.32*)
Is unfaithful	2.05 (1.59)	*ns*	1.44 (1.11-1.87)	*ns*	1.68 (1.29-2.18)	*1.29 (1.00-1.66*)	1.54 (1.29-1.85)	*1.30 (1.07-1.59*)	1.56 (1.25-1.94)	*ns*

* omitted from model as inclusion resulted in a failure to converge.

**Table 3:** Demographic predictors of IPV justification

	**Geography (Urban referent)**		**Age (25–34 referent)**				**Marital status (Not currently referent)**	
	**Rural**		**15-24**		**35+**		**Currently**	
	**Crude**	***Adjusted***	**Crude**	***Adjusted***	**Crude**	***Adjusted***	**Crude**	***Adjusted***
Any	1.75 (1.42-2.15)	*ns*	1.22 (1.00-1.48)	*ns*	1.19 (1.01-1.40)	*1.23 (1.04-1.44*)	1.98 (1.38-2.84)	*1.87 (1.30-2.67*)
Out w/out permission	ns	*ns*	ns	*	1.85 (1.09-3.13)	*	ns	*5.02 (1.08-23.22*)
Neglects kids	2.30 (1.38-3.85)	*ns*	ns	*ns*	ns	*ns*	ns	*ns*
Argues back	2.48 (1.22-5.03)	*ns*	ns	*ns*	ns	*ns*	ns	*ns*
Refuses sex	3.96 (1.05-14.89)	*ns*	2.05 (1.14-3.70)	*	ns	*	ns	*ns*
Housework/Food prep	4.61 (1.12-18.98)	*ns*	ns	*ns*	ns	*ns*	ns	*ns*
Ask about girlfriends	ns	*ns*	ns	*ns*	2.41 (1.53-3.81)	*2.85 (1.81-4.49*)	ns	*ns*
Is unfaithful	1.75 (1.42-2.15)	*ns*	ns	*ns*	1.20 (1.02-1.42)	*1.25 (1.05-1.48*)	2.10 (1.47-3.02)	*1.96 (1.35-2.85*)

* omitted from model as inclusion resulted in a failure to converge.

**Table 4:** Associations between experiences with domestic violence and IPV justification

	**Parental IPV**		**Child abuse**		**Partner abuse**		**Past year**	
	**(No = referent)**		**(No = referent)**		**(No = referent)**			
					**Prior to past year only**			
	**Crude**	**Adjusted**	**Crude**	**Adjusted**	**Crude**	**Adjusted**	**Crude**	**Adjusted**
Any*	1.73 (1.40-2.14)	*ns*	1.98 (1.60-2.45)	*1.64 (1.28-2.10*)	ns	*ns*	ns	*ns*
Out w/out permission	4.43 (2.40-8.17)	*2.17 (1.04-4.56*)	5.64 (3.12-10.19)	*2.17 (1.04-4.56*)	3.04 (1.38-6.70)	*3.43 (1.79-6.58*)	*ns*	*ns*
Neglects kids	2.60 (1.77-3.83)	*1.61 (1.00-2.58*)	3.26 (2.21-4.80)	*1.98 (1.33-2.98*)	2.34 (1.37-4.01)	*2.18 (1.31-3.62*)	ns	*ns*
Argues back	*2.75 (1.73-4.39*)	*1.86 (1.04-3.35*)	*2.94 (1.66-5.21*)	*ns*	ns	*ns*	ns	*ns*
Refuses sex	4.17 (2.50-6.97)	*2.09 (1.10-4.00*)	5.45 (2.98-9.94)	*2.46 (1.45-4.17*)	3.36 (1.72-6.54)	*3.30 (1.51-7.23*)	ns	*ns*
Housework/Food prep	4.84 (2.43-9.61)	*	7.79 (4.15-14.64)	*4.61 (2.54-8.36*)	4.41 (2.08-9.37)	*3.23 (1.55-6.73*)	3.20 (1.19-8.62)	*ns*
Ask about girlfriends	4.72 (2.74-8.13)	*2.72 (1.41-5.23*)	4.48 (2.58-7.77)	*2.29 (1.29-4.05*)	ns	*ns*	ns	*ns*
Is unfaithful	1.69 (1.36-2.11)	*ns*	1.97 (1.58-2.47)	*1.67 (1.29-2.15*)	ns	*ns*	ns	*ns*

* omitted from model as inclusion resulted in a failure to converge.

Table 3 explores the relationships between the socio-demographic factors of geography, age and marital status on a Georgian woman’s likelihood to justify IPV. A consistent pattern emerges that women from rural communities have an increased (two to up to five times) likelihood to justify IPV depending on the scenario. In terms of age, it appears that younger women (under 25) are more likely to justify IPV when a woman refuses sex when compared with women 25–34 but under no other circumstance. Alternatively, older women are more likely to justify IPV around issues of infidelity or going out without permission compared to the middle age group. As described above, only “ever married” women were asked questions regarding IPV justification in this survey. There are two circumstances where a currently married woman is significantly more likely to justify IPV compared with a woman who is divorced, separated or widowed: when a woman goes out without permission or is unfaithful.

Table 4 explores the role of past and current exposure to domestic violence on a Georgian woman’s justification of IPV. Consistently, women who experienced violence in their homes while growing up, in the form of parental IPV or child abuse, show an increased likelihood of supporting IPV compared to those women who did not experience this violence. Furthermore, having been abused as a child is consistently a strong predictor of current justification of IPV among this sample. Similarly, those women who experienced IPV prior to the past year were more likely to justify this behavior across four of the seven scenarios. Obtaining significance for past year IPV was challenging given the low prevalence of this among the sample. However, the unadjusted estimates reveal the currently abused women show an approximately a three-time increased likelihood to justify IPV when a woman fails in her household duties, as compared to non-currently abused women.

## Discussion

Georgian women report comparatively lower rates of IPV and lower justification of this behavior. For example, when looking at the closest bordering countries where data was available, in the second most common scenario where IPV was justified, neglect of a child, fewer than 5% of Georgian women felt this scenario justified IPV as compared to 26% of the women in Kazakhstan and 27% of the women in Armenia [29]. However, as found elsewhere, these two factors, victimization and justification of this behavior are strongly associated in this population. This association may lend insight to the lower levels of IPV found in Georgia; the lower tolerance of this behavior may act as a social deterrent even without strong legislation.

As noted in other countries [26,30,31], Georgian women who were poor, from rural areas, had lower education and were not working had the greatest likelihood to justify partner violence. Prior research restricted to males only found conflicting findings in terms of the association between IPV justification and educational levels [32]. However, in this study the two opposing samples also differed by age, marital status, education and potentially the content of and character of the educational system and the endorsement of patriarchal ideals. These findings support the WHO results and others identifying that lower socio economic status influence social perceptions toward partner violence. Lastly, while it was difficult to ascertain the impact of current IPV on justification given the small number of recently victimized women in this sample, experiences of violence either as a partner, a child or witness to IPV among parents increased the likelihood that a Georgian woman would justify this behavior. While past research has identified that increased justification is associated with increased risk of IPV [30], contextualizing the influence of abuse felt or witnessed during childhood further illuminates the influence of experienced violence on perceptions of acceptability.

In this cross-sectional survey the temporal order of IPV victimization and IPV justification could not be established. However, conceptually these two factors likely have a dynamic relationship with each impacting the other. As an analysis of secondary data, this study was limited to crude measures of physical violence with no contextual factors. Furthermore, the original researchers excluded never married women for the IPV justification questions. A small body of research has found that younger women are up to twice as likely to justify IPV compared to older women [13,33-37]. Whereas primarily the younger women fall into the “never married” category, a comparative analysis is not possible here. Other methodological issues included the decision to ask only those women who reported that they were raised in a household by both parents were asked if they witnessed IPV among their parents growing up. While, in the US, the high degree of IPV among divorced and separated parents is recognized, the in Georgia this familial experience (with or without violence) is rare. For our sample, with only 24 women overall skipped (less than 1%) there is little reason to expect this skip to have biased our findings. Lastly, as a multi-country study, situational measures for justification were predefined to allow international comparisons by the WHO. It is possible that scenarios developed specifically for this population would have revealed the behaviors that show stronger justification of abuse. Further research on this relationship applying culturally specific measures is warranted.

## Conclusions

The Republic of Georgia has experienced both recent and threated conflicts, which conceivably would increase the level of IPV [6] however, given that IPV justification is more likely historic, based largely on social norms, there is little reason to believe that justification of IPV is impacted by these conflicts. In Georgia, violence against women is historically unacceptable; with extended families often living together and strong family social support, it is difficult to keep this behavior secret. Perhaps, this social disapproval counteracts the impact of living in a state of conflict. Lastly, as found in other countries [12], the most consistent predictors of IPV justification among this sample were lower socio-economic status, lower education, living in a rural area and having personal experiences with domestic violence. These findings again, provide insight to the higher level of IPV victimization among these multi-marginal populations that lack a social barrier against the abuse of women by their intimate partners.

It is has been recognized that the justification of IPV directly influences both our ideas about who is to blame and our definition of the behaviors that identify this problem as a public health and a legal issue. Furthermore, partner violence justification can reveal important information about the perpetration, victimization and response to this type of violence in a specific region. As the Georgian society moves toward a more Western society-- teens and young women are becoming more sexually active before marriage and with a growing economy it will be easier for young couples to live on their own -- it is likely that risks will also move, albeit slowly. Georgia may serve as a case example that as historic protections are loosened (extended family living together, women being only sexually active with their husband) there will be a growing need to improve other types of protection, in this case education relative to the welfare of women. Thus, as Georgia becomes more “modern” changing attitudes about justification for IPV may become more important to prevent increases in IPV among this population.

## Consent

Written informed consent was obtained from the participants for the publication of this report and any accompanying images.

## Abbreviations

DRH/CDC: Division of Reproductive Health, Centers for Disease Control and Prevention; GERHS10: Women’s Reproductive Health Survey in Georgia; IPV: Intimate Partner Violence; MoLHSA: Georgian Ministry of Labor, Health and Social Affairs () in collaboration with the; NCDC: Georgian National Center for Disease Control; RHS: Women’s Reproductive Health Survey; UNFPA: United Nations Population Fund; USAID: United States Agency for International Development; WHO: World Health Organization.

## Competing interests

The authors declare that they have no competing interests.

## Authors’ contributions

EW contributed to the conceptualization, design, analysis and interpretation. MB contributed to the conceptualization, design, acquisition of data and interpretation. NA contributed to the acquisition of data and interpretation. SS contributed to the analysis and interpretation. LAM contributed to the conceptualization, design, analysis and interpretation. All authors contributed to draft, revisions and give final approval to this manuscript.

## Authors’ information

Eve Waltermaurer and Maia Butsashvili are co-first authors.

## Pre-publication history

The pre-publication history for this paper can be accessed here:

http://www.biomedcentral.com/1472-6874/13/44/prepub
